# Sex-Specific Equations to Estimate Maximum Oxygen Uptake in Cycle Ergometry

**DOI:** 10.5935/abc.20150089

**Published:** 2015-10

**Authors:** Christina G. de Souza e Silva, Claudio Gil S. Araújo

**Affiliations:** Programa de Pós-Graduação em Cardiologia - Universidade Federal do Rio de Janeiro; Instituto do Coração Edson Saad - Universidade Federal do Rio de Janeiro; Clínica de Medicina do Exercício - CLINIMEX, Rio de Janeiro, RJ - Brazil

**Keywords:** Breathing Exercise / utilization, Physical Exertion, Oxygen Consumption, Cardiopulmonary Exercise Testing, Demographic Data, Ergometry

## Abstract

**Background:**

Aerobic fitness, assessed by measuring VO_2_max in maximum cardiopulmonary exercise testing (CPX) or by estimating VO_2_max through the use of equations in exercise testing, is a predictor of mortality. However, the error resulting from this estimate in a given individual can be high, affecting clinical decisions.

**Objective:**

To determine the error of estimate of VO_2_max in cycle ergometry in a population attending clinical exercise testing laboratories, and to propose sex-specific equations to minimize that error.

**Methods:**

This study assessed 1715 adults (18 to 91 years, 68% men) undertaking maximum CPX in a lower limbs cycle ergometer (LLCE) with ramp protocol. The percentage error (E%) between measured VO_2_max and that estimated from the modified ACSM equation (Lang et al. MSSE, 1992) was calculated. Then, estimation equations were developed: 1) for all the population tested (C-GENERAL); and 2) separately by sex (C-MEN and C-WOMEN).

**Results:**

Measured VO_2_max was higher in men than in WOMEN: -29.4 ± 10.5 and 24.2 ± 9.2 mL.(kg.min)^-1^ (p < 0.01). The equations for estimating VO_2_max [in mL.(kg.min)^-1^] were: C-GENERAL = [final workload (W)/body weight (kg)] x 10.483 + 7; C-MEN = [final workload (W)/body weight (kg)] x 10.791 + 7; and C-WOMEN = [final workload (W)/body weight (kg)] x 9.820 + 7. The E% for MEN was: -3.4 ± 13.4% (modified ACSM); 1.2 ± 13.2% (C-GENERAL); and -0.9 ± 13.4% (C-MEN) (p < 0.01). For WOMEN: -14.7 ± 17.4% (modified ACSM); -6.3 ± 16.5% (C-GENERAL); and -1.7 ± 16.2% (C-WOMEN) (p < 0.01).

**Conclusion:**

The error of estimate of VO_2_max by use of sex-specific equations was reduced, but not eliminated, in exercise tests on LLCE.

## Introduction

Aerobic fitness is an independent predictor of mortality ^1-3^ and provides relevant diagnostic and prognostic information^4-8^. It is non-invasively assessed by measuring maximum oxygen uptake (VO_2_max) during exercise testing, in which expired gases are collected and analyzed. This procedure is called maximum cardiopulmonary exercise testing (CPX)^9,10^.

Although available at several clinical exercise testing laboratories, VO_2_max measurement requires professional training^11^ and specific equipment, and increases the time for test performance, hindering the wider use of CPX.

When CPX cannot be performed, VO_2_max can be estimated by use of equations based on duration^12^ or intensity at peak exertion^13,14^. By applying these equations to groups of individuals, the association between estimated and measured VO_2_max values tends to be good. However, the margin of error of estimate (EE) for a given subject can be large, greater than 15%^15^. Errors of such magnitude are rarely accepted in other biological variables, and exceed those observed in laboratory tests or in clinical and anthropometric measurements (height and weight). Considering that small variations in VO_2_max can lead to important differences in clinical management or sports training guidance^16^, such errors can be challenging, requiring some effort to minimize them.

Theoretically, the mechanical efficiency in performing a certain motor gesture is expressed by the ratio between the work generated and the oxygen consumed in its performance^17^. That efficiency varies between individuals and depends on age, sex, clinical condition and physical fitness. Most equations available for estimating VO_2_max, however, do not consider those possible relationships, which might contribute to errors in VO_2_max estimate. For example, considering anthropometric, physiological and biomechanical differences, as well as sports performance, the influence of sex on the EE of VO_2_max is worth assessing.

The objectives of this study were: a) to determine the EE of VO_2_max in cycle ergometry for a population undergoing CPX at a clinical exercise testing laboratory; and b) to propose sex-specific equations aimed at reducing the EE of aerobic capacity in cycle ergometry.

## Methods

### Sample

This study reviewed data of patients voluntarily submitted to CPX between January 2008 and June 2014 at a private clinical exercise testing laboratory. Patients simultaneously meeting the following inclusion criteria were selected: a) no previous assessment at the private clinical exercise testing laboratory; b) age ≥ 18 years; and c) maximum CPX performed on a lower limbs cycle ergometer (LLCE) (Inbrasport CG-04, Inbrasport, Brazil).

**Figure 1:**
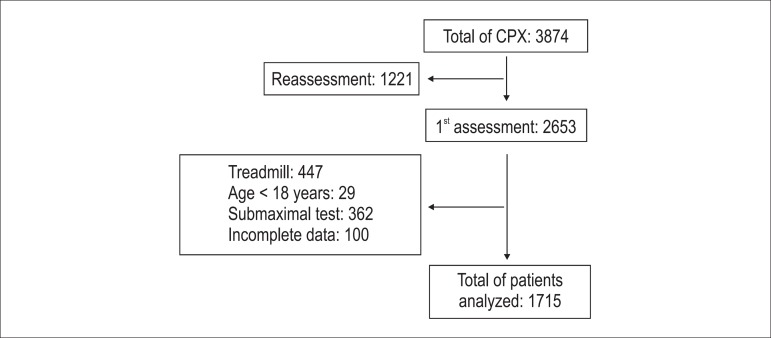
Flowchart of study sample selection.

During that period, 3874 assessments were performed and, after applying the inclusion criteria, 1715 individuals (1172 men) were included (Figure 1). In addition, 200 individuals subsequently undergoing CPX and meeting the inclusion criteria were used to validate the equations developed.

### Ethical considerations

All patients provided written informed consent before undergoing CPX. The retrospective analysis of data was approved by the Committee on Ethics and Research of the institution.

### Clinical assessment and body weight and height measurements

Before performing CPX, clinical history was taken, with emphasis on regularly used medications and cardiovascular risk factors, and physical examination was undertaken. Body weight and height of all individuals were measured. The prescribed medications were not suspended before CPX.

Body weight was measured with a Cardiomed scale, Welmy model, with 0.1-kg resolution. Height was measured with a Sanny stadiometer with 0.1-cm resolution.

### Maximum cardiopulmonary exercise testing

The CPX was conducted in a specific room, with temperature ranging from 21°C to 24°C, and relative air humidity between 40% and 60%. The test was performed according to an individualized ramp protocol, aimed at 8-12-minute duration, on an LLCE, according to the Brazilian Society of Cardiology guidelines ^18^, in the presence of a qualified physician, at a laboratory properly equipped to manage occasional clinical events. Only four physicians performed all the tests, following a routine of well-defined procedures, especially regarding the stimulus to reach truly maximum exertion. The height of the saddle was individually adjusted to provide both an almost complete knee extension at the lowest pedal position, and a lower-hip 90-degree flexion at the highest pedal position. The pedaling frequency was kept between 65 and 75 rotations per minute.

During CPX, the individuals were monitored with a digital electrocardiograph (ErgoPC Elite, versions 3.2.1.5 or 3.3.4.3 or 3.3.6.2, Micromed, Brazil), and heart rate (HR) was measured on the ECG recording (leads CC5 or CM5) at the end of each minute. Expired gases were collected by use of a Prevent pneumotacograph (MedGraphics, USA) coupled to a mouthpiece, with concomitant nasal occlusion. The expired gases were measured and analyzed by using a VO_2000_ metabolic analyzer (MedGraphics, USA), daily calibrated before the first assessment and whenever necessary. The mean results of the expired gases were read every 10 seconds, and consolidated at every minute. The highest VO_2_ value obtained at a certain point of the CPX was considered the VO_2_max. Blood pressure was measured every minute on the right arm by using a manual sphygmomanometer.

The maximum intensity of the exercise, which is more easily assessed by using CPX - presence of anaerobic threshold and U-pattern curves of ventilatory equivalents -, was confirmed by maximum voluntary exhaustion (score 10 in the Borg scale ranging from 0 to 10^19^) represented by the incapacity to continue pedaling at the previously established frequency despite strong verbal encouragement. As already reported in a previous study^20^, the characterization of CPX as maximum was also confirmed by the impression of the physician in charge, and recorded on the CPX description. It is worth noting that CPX was neither interrupted nor considered maximum based exclusively on HR.

### Equations to predict VO_2_max and maximum HR

The predicted values of VO_2_max for each patient, as a mere reference for comparison with the actually measured VO_2_max values, were obtained based on specific equations for men [60 - 0.55 x age (years)] and women [48 - 0.37 x age (years)]^21^.

The predicted values of maximum HR were obtained from the equation 208 - 0.7 x age^22^, for patients of both sexes.

### Equations to estimate VO_2_max

To assess the EE of VO_2_max, VO_2_max was initially estimated based on the modified American College of Sports Medicine (ACSM) equation^14^, in which VO_2_max is adjusted for body weight [mL.(kg.min)^-1^] as follows: (W x 11.4 + 260 + body weight x 3.5)/weight. In that equation, W is the maximum workload in watts, body weight is expressed in kg, and the constant 260 mL.min^-1^ represents the oxygen volume in mL and corresponds to the necessary energetic expenditure to pedal without any additional resistance [approximately 3.5 mL.(kg.min)^-1^ x mean body weight of the individuals studied by Lang et al.]^14^. In addition, the last term in that equation corresponds to the energetic expenditure at rest. Following that line of thought, and in accordance with that adopted by the ACSM^23^, in our study, we subtracted 7 mL.(kg.min)^-1^ from the VO_2_max value measured [corresponding to 3.5 mL.(kg.min)^-1^ of VO_2_ at rest and 3.5 mL.(kg.min)^-1^ of VO_2_ expended to pedal without any load]. The result obtained was divided by the ratio between workloads (watts) and body weight (kg), originating the constant “k” for each participant. From the mean value of the constant “k”, we obtained the multiplying factor values of the workloads (watts)/body weight (kg) ratio for the equations for the general sample, men and women, respectively: a) general equation to estimate VO_2_max (equation C-GENERAL); b) specific equation to estimate VO_2_max in the male sex (equation C-MEN); and c) specific equation to estimate VO_2_max in the female sex (equation C-WOMEN).

### Error of estimate of VO_2_max

The magnitude of the EE of VO_2_max expressed as a function of body weight was assessed based on the calculation of: 1) the difference between the measured and estimated values: (measured VO_2_max - estimated VO_2_max) in mL.(kg.min)^-1^; and the percentage error (E%): [(measured VO_2_max - estimated VO_2_max)/measured VO_2_max] x 100. The measured VO_2_max was obtained by collecting and analyzing expired gases, as previously detailed. A negative EE or E% value thus means that the estimated VO_2_max was higher than the measured VO_2_max, that is, the value calculated by using the equation overestimated the value measured.

### Statistical analysis

The results were expressed as mean and standard deviation or as percentage, depending on the nature of the variable. The demographic characteristics and CPX results were compared between men and women by using non-paired *t* test or chi-square test. The ER and E% of the equations, when appropriate, were compared by using paired *t* test or ANOVA, when the comparison was performed between three or more groups. The measured VO_2_max value and that estimated based on the three equations of the study - C-GENERAL, C-MEN and C-WOMEN - were compared and analyzed by using linear regression and intraclass correlation. The statistical analyses were performed with the programs Prism 6 (GraphPad, USA) and SPSS 16 (SPSS, USA), adopting 5% as the significance level.

## Results

### Demographic and clinical characteristics of the sample

The sample was mostly formed by men (68.3%), with age ranging from 18 to 91 years, and 23.2% had a body mass index (BMI) ≥ 30 kg.m^-2^. Table 1 and 2 show other demographic and clinical data, as well as the prevalence of some risk factors for coronary artery disease, major morbidities and medications regularly used.

**Table 1:** Major demographic and morphofunctional characteristics of the sample (n = 1715)*

**Demographic characteristics**	**Men 1172 (68.3%)**	**Women 543 (31.7%)**
Age (years)	53 ± 15	51 ± 15
BMI (kg.m^-2^)	27.9 ± 4.2	25.3 ± 4.9
Weight (kg)	85.9 ± 14.8	66.9 ± 12.8
Height (cm)	175.3 ± 6.9	162.6 ± 6.5
Predicted VO_2_max [mL(kg.min)^-1^)]	30.7 ± 8.1	29.3 ± 5.5
Predicted maximum HR (bpm)	170.7 ± 10.3	172.6 ± 10.5

BMI: body mass index; HR: heart rate.

* values expressed as mean ± standard deviation.

**Table 2:** Major clinical characteristics of the sample and regularly used medications (n = 1715)*

	**Men (n = 1172)**	**Women (n = 543)**
**Morbidities**		
Systemic arterial hypertension	428 (36.5%)	114 (21.0%)
Dyslipidemia	496 (42.6%)	140 (25.8%)
Obesity	193 (16.5%)	61 (11.2%)
Diabetes *mellitus*	113 (9.6%)	29 (5.3%)
Coronary artery disease	249 (21.2%)	39 (7.2%)
Acute myocardial infarction	125 (10.7%)	18 (3.3%)
Myocardial revascularization	96 (8.2%)	10 (1.8%)
Use of medications		
Beta-blocker	302 (25.8%)	91 (16.8%)
Calcium channel blocker	109 (9.3%)	37 (6.8%)
ACEI	125 (10.7%)	19 (3.5%)
ARB	340 (29.0%)	113 (20.8%)
Diuretic	186 (15.9%)	68 (12.5%)
Vasodilator	82 (7.0%)	14 (2.6%)
Lipid-lowering	531 (45.3%)	151 (27.8%)
Antiplatelet	387 (33.0%)	82 (15.1%)
Antiarrhythmic	71 (6.1%)	25 (4.6%)

ARB: angiotensin-receptor blocker; ACEI: angiotensin-converting-enzyme inhibitor.

* values expressed as N(%).

### CPX data

The mean duration of CPX was 10 ± 2 minutes. The mean maximum HR for the set of individuals was 159 ± 25 bpm, corresponding to 92% of that predicted, being higher in patients not on beta-blockers (166 ± 20 bpm) (p < 0.01). Men achieved final workloads higher than women (172 ± 70 vs 111 ± 45 watts; p < 0.01), as well as greater VO_2_max values [29.4 ± 10.5 vs 24.2 ± 9.2 mL.(kg.min)^-1^; p < 0.01]. In the sample studied, the measured VO_2_max tended to be slightly lower than that predicted based on age and sex, corresponding to 96% and 82% of the value predicted by using the equations of Jones et al.^21^ for men and women, respectively. Table 3 shows the major CPX results.

**Table 3:** Major results of cardiopulmonary exercise test (n = 1715)*

**Variable**	**Men (n = 1172)**	**Women (n = 543)**
Duration (min)	10 ± 2	9 ± 3
Maximum HR (bpm)	158 ± 26	161 ± 24
- with beta-blocker	135 ± 25	133 ± 24
- without beta-blocker	166 ± 21	167 ± 20
Maximum workload (watts)	172 ± 70	111 ± 45
Measured VO_2_max [mL.(kg.min)^-1^)]	29.4 ± 10.5	24.2 ± 9.2

HR: heart rate.

* values expressed as mean ± standard deviation.

### Estimated VO_2_max values

Regarding estimated VO_2_max, the values obtained by using the modified ACSM equation were 29.8 ± 9.8 and 26.9 ± 8.9 mL.(kg.min)^-1^ for men and women, respectively, showing that the equation tends to overestimate VO_2_max. Both ER and E% differed between sexes (p < 0.01), with values of -0.4 ± 3.2 mL.(kg.min)^-1^ and -3.4 ± 13.4% for men, and -2.7 ± 3.5 mL.(kg.min)^-1^ and -14.7 ± 17.4% for women, respectively.

### C-GENERAL equation

Determining the specific equation for the sample studied, with no distinction between sexes and with the same variables of the modified ACSM equation, the following C-GENERAL equation was obtained: (final workload/body weight x 10.483 + 7, where 7, as previously explained, corresponds to a simplification of the last two terms of that equation [the addition of oxygen uptake at rest (3.5 mL.(kg.min)^-1^ and an identical oxygen uptake value to pedal with no resistance]. Applying the C-GENERAL equation, the estimated VO_2_max values obtained were 28.3 ± 8.9 mL.(kg.min)^-1^ and 24.9 ± 7.9 mL.(kg.min)^-1^ for men and women, respectively. Although EE and E% values remained similar in men [1.1 ± 3.3 mL.(kg.min)^-1^ and 1.2 ± 13.2%, respectively], a significant reduction in the EE of VO_2_max was observed in women [-0.7 ± 3.5 mL.(kg.min)^-1^], and E% was -6.3 ± 16.5% (p < 0.01).

### C-MEN and C-WOMEN equations

**Figure 2:**
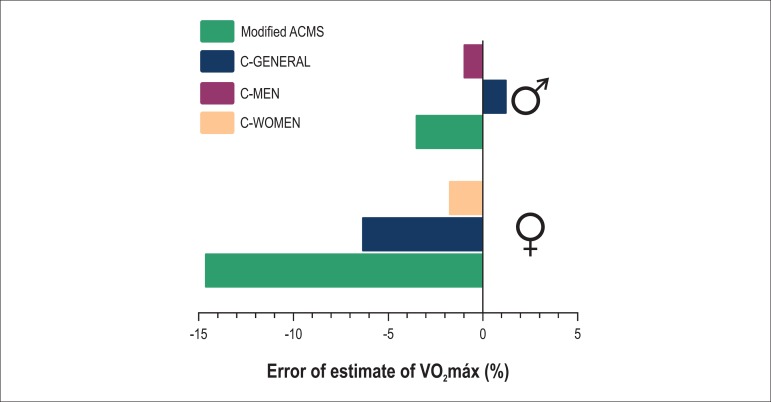
Percentage errors obtained from the comparison between measured VO2max and estimated VO2max by using the modified ACSM equations, and the C-GENERAL, C-MEN e C-WOMEN equations.

Then the following sex-specific equations, C-MEN and C-WOMEN were obtained: (final workload/body weight) x 10.791 + 7 and (final workload/body weight) x 9.820 + 7, respectively. Using these equations, the estimated VO_2_max values were 28.9 ± 9.2 mL.(kg.min)^-1^ and 23.7 ± 7.4 mL.(kg.min)^-1^ for men and women, respectively. Errors of estimate were reduced in both sexes, but more expressively for women. For men, EE and E% were 0.5 ± 3.2 mL.(kg.min)^-1^ and -0.9 ± 13.4% (p < 0.01), respectively, while for women, they were reduced to 0.5 ± 3.6 mL.(kg.min)^-1^ and only -1.7 ± 16.2% (p < 0.01), respectively (Figure 2).

**Figure 3:**
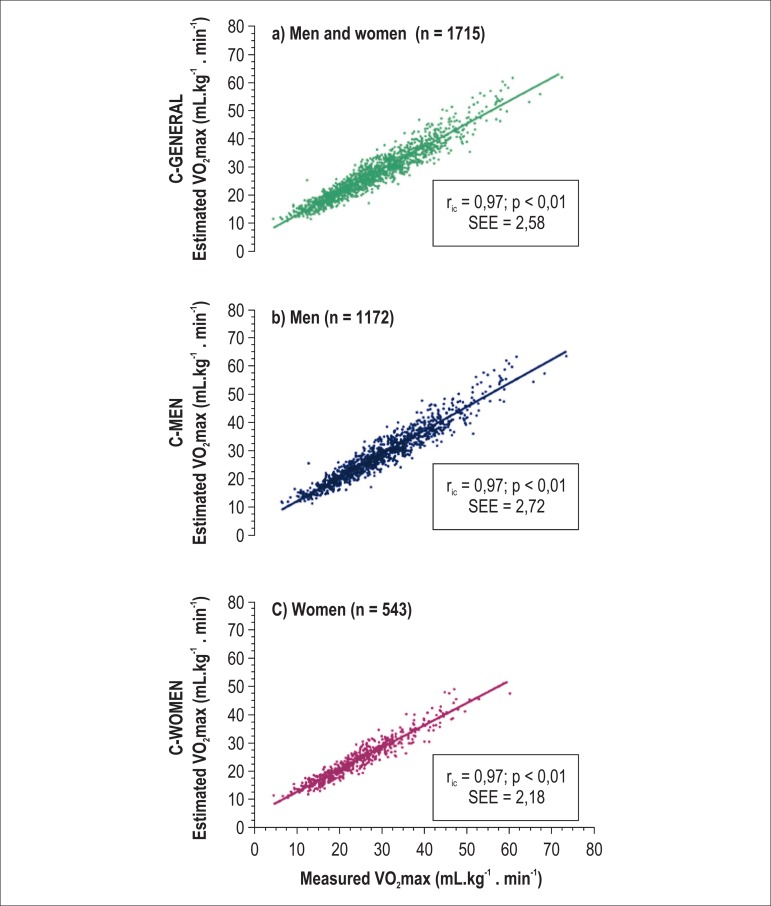
Correlation between measured VO_2_max values and those estimated by using the equations: a) C-GENERAL, b) C-MEN and c) C-WOMEN. SEE: standard error of estimate; r_ic_: intraclass correlation coefficient.

Figure 3 shows the standard EE and the association between the estimated and measured VO_2_max values for the general sample and for men and women, analyzed separately. It is worth noting the high intraclass correlation coefficients, with their respective confidence intervals (CI) obtained: C-GENERAL, 0.9703 (95%CI: 0.9674 - 0.9730); C-MEN, 0.9725 (95%CI: 0.9691 - 0.9755), and C-WOMEN, 0.9680 (95%CI: 0.9621 - 0.9729). The visual inspection of the distributions allowed characterizing the linear regressions as homoscedastic.

Based on the application of the equations developed in the present study, the following EE and E% were obtained in the validation sample: C-GENERAL (n = 200) 0.5 ± 2.5 mL.(kg.min)^-1^ and 0.7 ± 9.1%; C-MEN (n = 135) 0.5 ± 2.5 mL.(kg.min)^-1^ and 1.0 ± 8.6%; and C-WOMEN (n = 65) 0.5 ± 2.0 mL.(kg.min)^-1^ and 0.5 ± 8.5%, respectively.

## Discussion

The CPX is the most appropriate test to assess aerobic capacity. However, the use of the exercise test with neither collection nor analysis of expired gases is very common among us, even though accompanied by a significant margin of error ^15^. Therefore, it is important to develop specific equations to reduce that EE in exercise tests performed at hospitals and clinics.

Although previous studies with that same objective have been conducted^24-27^, the use of small samples hinders the extrapolation of the results found. For example, Lang et al.^14^ and Latin et al.^28^ have used the ACSM equation to estimate VO_2_max^13^ for 60 men and 60 women, respectively, and have found lower estimated VO_2_max values than the measured ones, for both sexes. On the other hand, Greiwe et al.^29^, applying that same equation to 15 men and 15 women with similar clinical profiles, have obtained overestimated VO_2_max values. In addition, the introduction by Lang et al.^14^ of the factor 260 mL.min^-1^, which corresponds to the energetic expenditure of pedaling without additional resistance, has produced estimated results more similar to measured VO_2_max results in their sample. In our study, however, the use of that modified ACSM equation maintained significant errors in the comparison between estimated and measured values. The discrepancy in the results described suggests significant errors when the equations are developed based on small samples.

In addition, the difference in EE between men and women using the same equation suggests that sex-specific equations should be developed. Storer et al.^30^ have developed three equations of to estimate VO_2_max using the variables workload, body weight and age: one general for both sexes; one specific for men; and one specific for women. Those authors have reported a significant increase in the coefficient of determination when the variable ‘sex' was added to the linear regression model used to create the equations. However, when applied to 77 men and 30 women of the Brazilian population^31^, a trend to overestimate VO_2_max was observed in men, evidencing the need to develop specific equations for each population.

Recently, Almeida et al.^32^ have conducted an important study with a large sample of Brazilians (3119 individuals), aimed at developing an equation to predict VO_2_max for treadmill exercise tests, based on age, sex, BMI and physical activity level. However, it is worth noting that, despite the importance of having VO_2_max reference data from equations developed for the Brazilian population, this does not contemplate the EE of VO_2_max when expired gases are not collected and analyzed during exercise testing. While the predicted VO_2_max is obtained based on pre-test clinical variables, such as age and sex, the estimated VO_2_max is calculated based on variables obtained during exercise testing, such as workload and test duration. To the best of our knowledge, there is no study on the Brazilian population with a large sample (more than 1000 cases) developing specific equations to estimate VO_2_max in exercise tests performed on a LLCE.

In reality, sample size and representability are extremely relevant. Neder et al.^33^ have observed that individuals typically selected to participate in studies did not represent those most commonly referred for exercise testing, which could lead to selection biases. Thus, in our study, we chose not to exclude obese patients, individuals with cardiovascular or pulmonary diseases and/or individuals on regular use of medications that could influence the physiological responses to exercise, to guarantee a sample representing the individuals most commonly referred to clinical exercise testing laboratories. It is worth noting that despite that varied clinical profile, the VO_2_max predicted for age was relatively close to that actually measured, especially in men. Comparing the data obtained in our study with those reported by Herdy and Uhlendorf^34^ in the Brazilian Southern region, the VO_2_max values measured in men were similar to the reference values for sedentary individuals aged 55 to 64 years [30.0 ± 6.3 mL.(kg.min)^-1^] or active individuals aged 65 to 74 years [30.0 ± 6.1 mL.(kg.min)^-1^]. The VO_2_max values found for women were similar to the reference values of sedentary individuals aged 55 to 64 years [23.9 ± 4.2 mL.(kg.min)^-1^]^34^. The most probable reason for that slight discrepancy is due to the fact that the study by Herdy and Uhlendorf^34^ used CPX on a treadmill, which might explain the tendency towards higher values for the same age group.

The strong points of our study are as follows: 1) to our knowledge, no other Brazilian study assessing equations for VO_2_max estimation was based on such a large number of individuals (over 1000); 2) the cycle ergometers and gas analyzers were periodically calibrated according to the specifications of their manufacturers; and 3) all original information of test reports was available in the digital format (data bank) and carefully reviewed to exclude those incomplete.

This study has limitations. All tests were performed following the ramp protocol. Thus, one cannot know if the equations for VO_2_max estimate here presented can be applied to exercise tests performed following other protocols.

Other factors, such as age, adiposity level, recent pattern or history of regular physical training, and use of certain medications, might contribute to the EE by influencing mechanical efficiency. This was a preliminary study to assess the influence of sex on the EE of VO_2_max. Other variables are being assessed, as already reported. Subsequent statistical analyses, such as multivariate regression, using the variables that evidenced influence on EE of VO_2_max can lead to the development of one single equation for VO_2_max estimate capable of effectively reducing EE.

Briefly, the present study contributed to current knowledge by proposing equations derived from a large sample of Brazilian adults, with clinical characteristics and profiles similar to those usually observed at clinical exercise testing laboratories. The equations are specific to the male and female sexes, thus contributing to reduce EE when VO_2_max measurement is not available.

## Conclusion

Our study identified that the use of foreign equations (modified ACSM) induced an important EE when applied to a typical population of clinical exercise testing laboratories in Brazil. Thus, an equation was developed - C-GENERAL -, partially reducing EE. However, an analysis separated by sex identified the need to develop specific equations - C-MEN and C-WOMEN - that could further reduce, but not eliminate, EE. Thus, more accurate alternatives to VO_2_max estimate in exercise tests of lower limbs are presented to places with no condition to effectively perform CPX to measure VO_2_max.
